# Yeast Bax Inhibitor, Bxi1p, Is an ER-Localized Protein That Links the Unfolded Protein Response and Programmed Cell Death in *Saccharomyces cerevisiae*

**DOI:** 10.1371/journal.pone.0020882

**Published:** 2011-06-06

**Authors:** James Cebulski, Joshua Malouin, Nathan Pinches, Vincent Cascio, Nicanor Austriaco

**Affiliations:** Department of Biology, Providence College, Providence, Rhode Island, United States of America; University of Washington, United States of America

## Abstract

Bax inhibitor-1 (BI-1) is an anti-apoptotic gene whose expression is upregulated in a wide range of human cancers. Studies in both mammalian and plant cells suggest that the BI-1 protein resides in the endoplasmic reticulum and is involved in the unfolded protein response (UPR) that is triggered by ER stress. It is thought to act via a mechanism involving altered calcium dynamics. In this paper, we provide evidence that the *Saccharomyces cerevisiae* protein encoded by the open reading frame, YNL305C, is a *bona fide* homolog for BI-1. First, we confirm that yeast cells from two different strain backgrounds lacking YNL305C, which we have renamed *BXI1*, are more sensitive to heat-shock induced cell death than wildtype controls even though they have indistinguishable growth rates at 30°C. They are also more susceptible both to ethanol-induced and to glucose-induced programmed cell death. Significantly, we show that Bxi1p-GFP colocalizes with the ER localized protein Sec63p-RFP. We have also discovered that *Δbxi1* cells are not only more sensitive to drugs that induce ER stress, but also have a decreased unfolded protein response as measured with a UPRE-lacZ reporter. Finally, we have discovered that deleting *BXI1* diminishes the calcium signaling response in response to the accumulation of unfolded proteins in the ER as measured by a calcineurin-dependent CDRE-lacZ reporter. *In toto,* our data suggests that the Bxi1p, like its metazoan homologs, is an ER-localized protein that links the unfolded protein response and programmed cell death.

## Introduction

Bax inhibitor-1 (BI-1) was first cloned in mammals from adult testes of different species, as the testis enhanced gene transcript (TEGT). [1], [2], [3], [4] However, its anti-apoptotic function was identified several years later through a functional yeast screen designed to select for human cDNAs that inhibit BAX induced apoptosis. [5] Importantly, BI-1 also suppressed apoptosis induced by BAX in human 293 kidney cells. [6]

The anti-apoptotic function of BI-1 contributes to the development of cancer: BI-1 expression is upregulated in human breast cancer, prostate cancer, brain tumors, large cell lymphoma, and cervical cancer. [7], [8], [9], [10], [11], [12], [13] In contrast, down-regulation of the gene is associated with the progression of chronic liver disease.[14] More recent work has also revealed that overexpressing BI-1 not only transforms NIH3T3 cells but also enhances cancer metastasis by altering glucose metabolism and activating the sodium-hydrogen exchanger. [13], [15] Finally, two groups have shown that overexpression of the protein protects animals from obesity-associated insulin resistance [16] and stroke and traumatic brain injury. [17]

The BI-1 protein has several trans-membrane domains and resides in the endoplasmic reticulum. [6], [18], [19] Cells isolated from BI-1^−/−^ mice exhibited ER stress-induced hypersensitivity suggesting that the protein is involved in ER function and the unfolded protein response (UPR). [19] The UPR is an integrated signal transduction pathway that transmits information about the protein-folding status in the ER lumen to the cytosol and the nucleus to increase protein-folding capacity. [20] If the UPR remains unresolved or prolonged, this will result in ER Ca^2+^ release promoting apoptosis. Mechanistically, several reports have proposed that BI-1 acts to inhibit ER stress signaling pathways. [19], [21] For example, Lee et al. have demonstrated that BI-1 can interfere with all three ER signaling pathways found in mammalian cells. [22] BI-1 has also been found to negatively regulate IRE1 activity, where IRE1 is a highly conserved ER stress transducer found in yeast, flies, and mice, that modulates the UPR. [23] Finally, in mammalian cells, BI-1 appears to regulate the concentration of Ca^2+^ in the endoplasmic reticulum and the cytosol. [24] Two papers suggest that BI-1 can act as an ER membrane Ca^2+^/H^+^ antiporter allowing Ca^2+^ release into the cytoplasm. [24], [25] Together, the data supports the proposal that mammalian BI-1 controls apoptosis by regulating calcium homeostasis.

Bax inhibitor-1 is a highly conserved protein with homologs found in *Arabidopsis thaliana, Drosophila melanogaster, Saccharomyces cerevisiae*, and *Escherichia coli*, among others. [26] Plant BI-1 from is able to suppress mammalian BAX induced cell death in yeast, 293 kidney cells, and plants. [6], [27], [28], [29], [30] Arabidopsis BI-1 has also been linked to an ER stress-mediated programmed cell death response. [31] *Drosophila* Bax inhibitor-1 too has similar functions. [18] In contrast, there is conflicting data regarding the function of the putative yeast Bax inhibitor-1 homolog encoded by the *Saccharomyces cerevisiae* open reading frame, YNL305C. One study has shown that it is able to suppress mammalian BAX induced, heat shock induced, and hydrogen peroxide induced cell death in yeast. [18] However, another report has also concluded the yeast protein encoded by YNL305C may not be a close ortholog of Bax inhibitor-1, because it is not involved in the unfolded protein response (UPR). [23]

In this paper, we provide evidence that suggests that the *Saccharomyces cerevisiae* protein encoded by YNL305C is a *bona fide* member of the Bax Inhibitor superfamily. First, we confirm that yeast cells from two different strain backgrounds lacking YNL305C, which we have renamed *BXI1*, are relatively more susceptible to heat-shock than wildtype controls. Though they have similar growth rates to their wildtype counterparts at 30°C, cells carrying a null mutation in *BXI1* are more sensitive both to ethanol-induced and to glucose-induced programmed cell death. Significantly, we show that Bxi1p-GFP colocalizes with the ER localized protein, Sec63p-RFP, suggesting that yeast Bax inhibitor-1 functions in the endoplasmic reticulum like its mammalian counterparts. Next, we discovered that *Δbxi1* cells are not only more sensitive to drugs that induce ER stress, but also have a decreased unfolded protein response (UPR) as measured with a UPRE-lacZ reporter. Finally, we have discovered that deleting *BXI1* diminishes the calcium signaling response in response to the accumulation of unfolded proteins in the ER as measured by a calcineurin-dependent CDRE-lacZ reporter. *In toto,* our data suggests that Bxi1p, like its eukaryotic homologs, is an ER-localized protein that links the UPR and programmed cell death in yeast.

## Materials and Methods

### Yeast Strains, Plasmids, and Growth Conditions

All experiments were done with isogenic *Saccharomyces cerevisiae* strains in either the BY4742 background (MATα *his3Δ1, leu2Δ0, lys2Δ0, ura3Δ0*) or the Σ1278b background (MATα *ura3Δ leu2Δ his3Δ::hisG*). The BY4742*Δbxi1* and the Σ1278b*Δbxi1* mutants were obtained from Open Biosystems (Huntsville, AL) and the Boone Laboratory (University of Toronto) [32] respectively. A yeast strain (ATCC 201388: MAT**a** *his3Δ1, leu2Δ0, met15Δ0, ura3Δ0*) containing a chromosomally tagged GFP-tagged Bxi1p [33] was purchased from Invitrogen. Plasmid pSM1960, a high copy (2 µ URA3 plasmid expressing Sec63p-RFP [34], was generously provided by Susan Michaelis (Johns Hopkins University). Plasmid pMCZ-Y, a high copy (2 µ URA3 plasmid containing a UPRE-lacZ reporter [35], was generously provided by David Eide (University of Wisconsin). Plasmid pAMS366, a high copy (2 µ URA3 plasmid containing a 4XCDRE-lacZ reporter [36], was generously provided by Kyle Cunningham (Johns Hopkins University). For all the experiments described in this paper, cells were cultured and treated using standard yeast protocols. [37] Unless noted otherwise, all drugs and reagents were purchased from SIGMA-Aldrich.

### Heat Shock Assay

Cells of the indicated genotype were cultured in rich YPD media overnight. They were then resuspended in fresh media and allowed to reach exponential phase (an approximate OD_600_ value of 0.4). Next, liquid cultures were heat shocked at 50°C in a water bath for the indicated times, serially diluted onto YPD plates, and cultured at 30°C for 2 days. At least three independent cultures were tested and compared. Statistical significance was determined with the Student's t-test.

### Ethanol-Induced Cell Death Assay

Cells of the indicated genotype were cultured in rich YPD media overnight, resuspended in fresh media, and allowed to reach exponential phase (an approximate OD_600_ value of 0.4). They were then resuspended in fresh media or in fresh media containing 22% ethanol [38], allowed to grow at 30°C for the indicated times, serially diluted onto YPD plates, and cultured at 30°C for 2 days. At least three independent cultures were tested and compared. Statistical significance was determined with the Student's t-test.

### Glucose-Induced Cell Death Assay

Cells of the indicated genotype were cultured in rich YPD media overnight, resuspended in fresh media, and allowed to reach exponential phase (an approximate OD_600_ value of 0.4). They were then resuspended in water or in water containing 2% glucose [39], [40], allowed to grow at 30°C for the indicated times, serially diluted onto YPD plates, and cultured at 30°C for 2 days. At least three independent cultures were tested and compared. Statistical significance was determined with the Student's t-test.

### Spot Assays

Cells were grown overnight in synthetic complete media at 30°C and then diluted to a final concentration (an approximate OD_600_ value of 0.4). For each strain, a series of 10-fold dilutions was then prepared in water over a range of concentrations from 10^−1^ to 10^−5^ relative to the initial culture. Spots of 5 µl from each dilution series were then plated on the indicated media and cultured at 30°C for 2 days. Plates supplemented with drugs were poured and used on the same day. All spot assays were repeated at least three times and a representative experiment is shown.

### Yeast β-galactosidase Assays

Cells of the indicated genotype were transformed with a plasmid containing the appropriate LacZ reporter, grown overnight in selective complete media at 30°C. Next, they were resuspended in fresh media and allowed to reach exponential phase (an approximate OD_600_ value of 0.4). They were then resuspended in fresh media or in fresh media containing drug, and allowed to grow at 30°C for three hours. β-galactosidase assays with the colorimetric substrate *o*-nitrophenyl-β-D-galactopyranoside (ONPG) were then performed using the permeabilized cell method and quantified as previously described. [37], [41], [42] Statistical significance was determined with the Student's t-test.

### Fluorescence Microscopy

Cells containing both GFP-Bxi1p and RFP-Sec63p were grown to mid-log phase in selective media and examined with a Zeiss LSM 700 Laser Confocal Microscope (Zeiss, Thornwood, NY), using a 63X oil-immersion objective and a pinhole size of 1 Airy Unit. Images were captured and processed using the Zen 2009 software package.

## Results and Discussion

Bax inhibitor-1 is a highly conserved mammalian protein with homologs found in *Arabidopsis thaliana, Drosophila melanogaster, Saccharomyces cerevisiae,* and *Escherichia coli,* among others. [26] However, there is conflicting data regarding the function of the putative yeast Bax inhibitor-1 homolog encoded by the *Saccharomyces cerevisiae* open reading frame, YNL305C. [18], [23] To resolve this discrepancy, we decided to investigate further the function of the yeast protein encoded by the open reading frame, YNL305C, which, in light of our findings, we have renamed *BXI1* for yeast Bax inhibitor-1.

**Figure 1:**
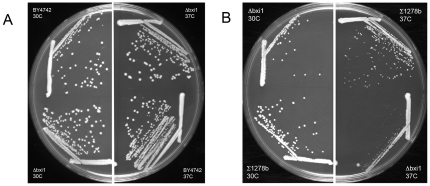
Wildtype and mutant *Δbxi1* cells have indistinguishable growth kinetics at 30°C. Wildtype and *Δbxi1* cells from the (A) BY4742 and (B) Σ1278b strain backgrounds were streaked on rich YPD plates and cultured at 30°C and at 37°C for two days.

**Figure 2:**
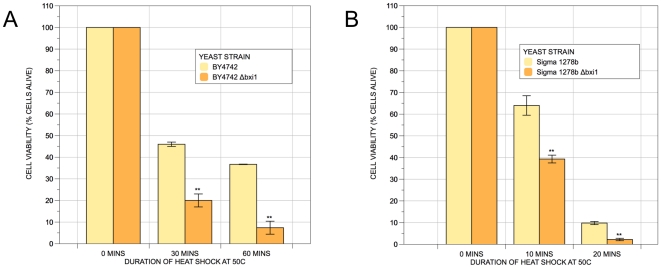
Yeast cells lacking *BXI1* are sensitive to heat shock. Wildtype and *Δbxi1* cells from the BY4742 and Σ1278b strain backgrounds were cultured in rich YPD media overnight. They were then resuspended in fresh media and allowed to reach exponential phase (an approximate OD_600_ value of 0.4). Liquid cultures were then heat shocked at 50°C in a water bath for the indicated times, serially diluted onto YPD plates, and cultured at 30°C for 2 days. The difference in viabilities was deemed statistically significant by the Student's t-test (p<0.05). Error bars indicate standard deviations for trials with at least three independent cultures.

We began by comparing the growth kinetics of wildtype and *Δbxi1* cells from two unrelated yeast strain backgrounds. Cells lacking *BXI1* are not slow growers: Their colony sizes were indistinguishable from those of wildtype cells on rich YPD media at 30°C (Figure 1). The growth rates of the wildtype and *Δbxi1* mutant cells from the BY4742 background were also comparable at 37°C (Figure 1A), though there was a difference in colony sizes in the Σ1278b strain background at the elevated temperature (Figure 1B). In general, the filamentous wildtype strain Σ1278b appears to be less robust than its BY4742 wildtype counterpart. We then turned to heat shock experiments to compare the phenotypes of wildtype and mutant cells in order to replicate published data. Chae et al. had discovered that cells lacking YNL305C were more sensitive to heat shock than controls. [18] As shown in Figure 2, we confirmed this result in both of our own strains suggesting that *BXI1* is involved in the yeast cell's response to heat stress induced cell death.

**Figure 3:**
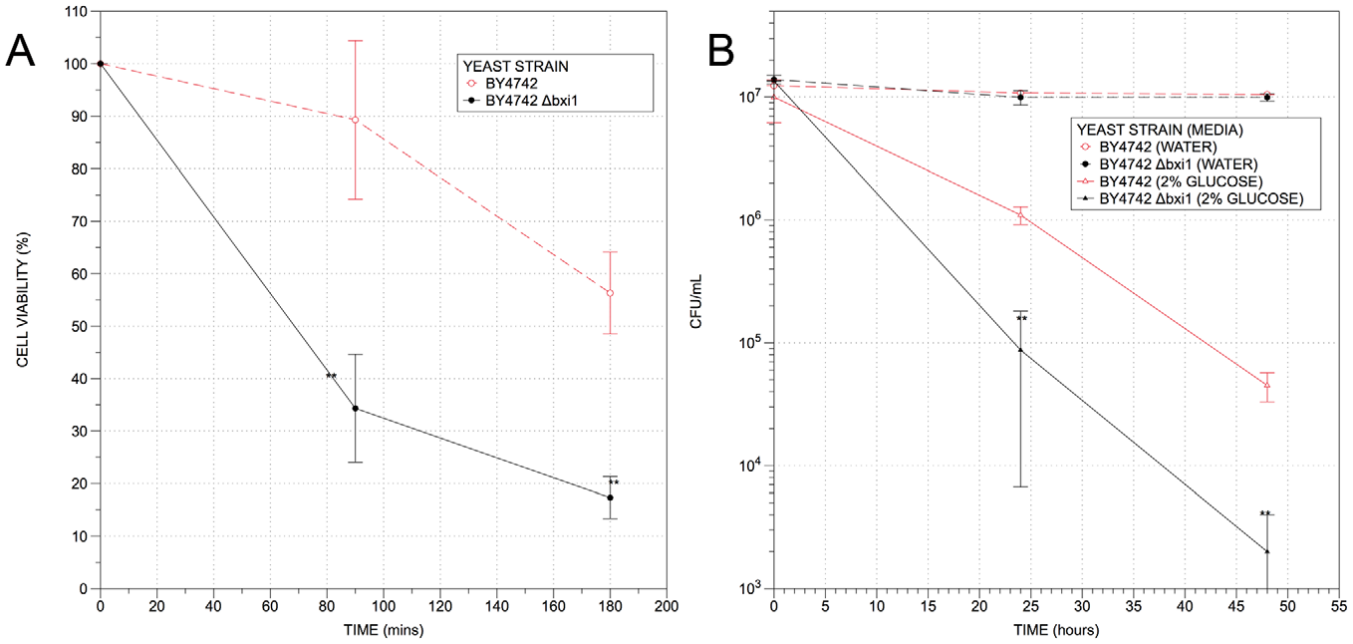
Yeast cells lacking *BXI1* are more susceptible to ethanol-induced and glucose-induced programmed cell death. Wildtype and *Δbxi1* cells from the BY4742 strain background were cultured in rich YPD media overnight and resuspended in fresh media and allowed to reach exponential phase (an approximate OD_600_ value of 0.4). (A) Exponential growing yeast cells were then resuspended in fresh media or in fresh media containing 22% ethanol, allowed to grow at 30°C for the indicated times, serially diluted onto YPD plates, and cultured at 30°C for 2 days. (B) Exponential growing yeast cells were then resuspended in water or in 2% glucose, allowed to grow at 30°C for the indicated times, serially diluted onto YPD plates, and cultured at 30°C for 2 days. The differences in viabilities were deemed statistically significant by the Student's t-test (p<0.05). Error bars indicate standard deviations for trials with at least three independent cultures.

Numerous studies have shown that mammalian and plant BI-1 proteins are involved in programmed cell death (PCD). [19], [21], [43], [44] Significantly both wildtype and mutant *Δbxi1* cells cultured in 15% ethanol for 1 hr contain reactive oxygen species (ROS) and fragmented mitochondria (data not shown), two markers of programmed cell death in yeast [45], [46], suggesting that deleting *BXI1* does not prevent yeast cells from undergoing PCD. To determine if *BXI1* is also involved in PCD in yeast, we cultured wildtype and *Δbxi1* cells in media containing 22% ethanol or in 2% glucose alone, two different conditions that have been shown to induce programmed cell death in yeast. [38], [39], [40] As shown in Figure 3, like their metazoan counterparts, yeast cells lacking *BXI1* were significantly more susceptible to these two different kinds of PCD than wildtype controls suggesting that the gene protects cells from programmed cell death.

**Figure 4:**
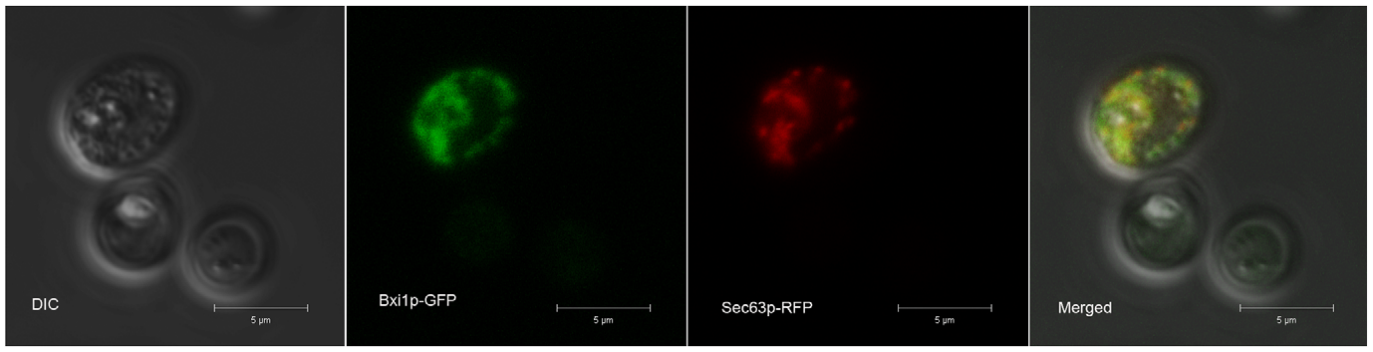
Yeast Bxi1p colocalizes with Sec63p, a marker for the endoplasmic reticulum. Wildtype cells containing both GFP-Bxi1p and RFP-Sec63p were grown to mid-log phase in selective media and examined with a Zeiss LSM 700 Laser Confocal Microscope using a 63X oil immersion objective and a pinhole size of 1 Airy Unit. This colocalization was seen in 74% of exponentially growing cells expressing both tagged proteins.

BI-1 is localized to the endoplasmic reticulum in both mammalian and plant cells. [6], [18], [19] To investigate the location of yeast Bxi1p, and to see if it too is localized to the ER, we obtained a yeast strain containing a chromosomally tagged GFP-tagged Bxi1p [33] from Invitrogen. The strain was neither sensitive to heat shock induced cell death nor sensitive either to β-mercaptoethanol (BME) or to tunicamycin (TM), suggesting that the GFP tag had not compromised the function of Bxi1p (data not shown). We then transformed the strain with a plasmid expressing RFP-Sec63p and imaged the cells with a laser confocal microscope. As shown in Figure 4, GFP-Bxi1p co-localizes with RFP-Sec63p, a protein that is found in the yeast endoplasmic reticulum membrane [32]. This colocalization was seen in 74% of exponentially growing cells expressing both tagged proteins. This suggests that yeast Bax inhibitor-1, Bxi1p, like mammalian and plant BI-1, functions in the endoplasmic reticulum. In a subset of the remaining cells, the GFP-Bxi1p appears to localize to the vacuole. [33]

**Figure 5:**
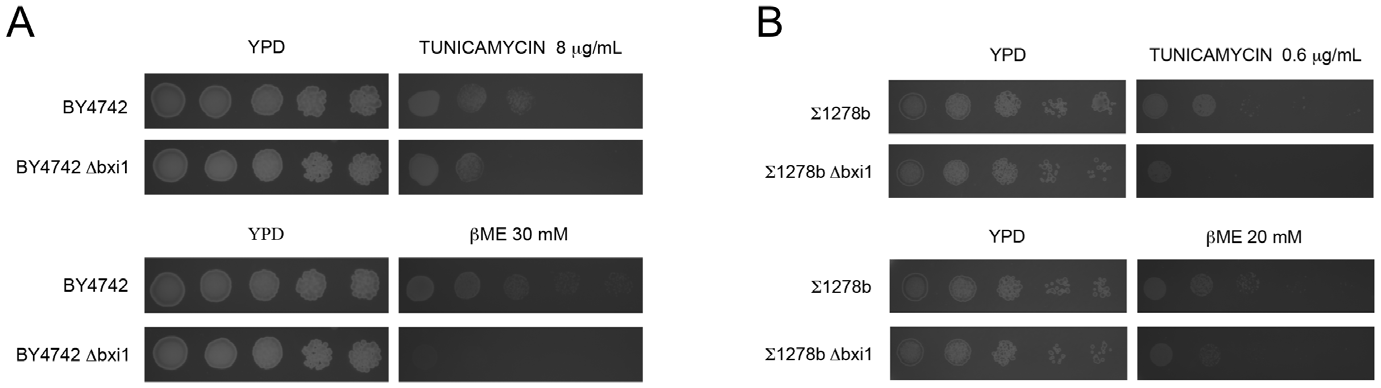
Yeast cells lacking *BXI1* are sensitive to β-mercaptoethanol and tunicamycin. Wildtype and *Δbxi1* cells from the BY4742 and Σ1278b strain backgrounds were cultured in rich YPD media overnight. They were then resuspended in fresh media and allowed to reach exponential phase (an approximate OD_600_ value of 0.4). For each strain, a series of 10-fold dilutions was then prepared in water over a range of concentrations from 10^−1^ to 10^−5^ relative to the initial culture. Spots of 5 µl from each dilution series were then plated on the indicated media and cultured at 30°C for 2 days. Plates supplemented with drugs were poured and used on the same day. All spot assays were repeated at least three times and a representative experiment is shown.

Given its ER localization, we predicted that *BXI1,* like its metazoan counterparts, may also be involved in the cell's response to ER stress. To test this hypothesis, we plated wildtype and *Δbxi1* cells from two unrelated yeast strain backgrounds onto plates containing β-mercaptoethanol (BME) and tunicamycin (TM), two drugs that are known to induce ER stress and the unfolded protein response. [19] Mechanistically, β-mercaptoethanol and tunicamycin cleave protein disulfide bonds, and block the synthesis of all N-linked glycoproteins, respectively. Our experiments revealed that *Δbxi1* yeast cells, regardless of strain background, are more sensitive to both these ER stress-inducing drugs suggesting that they were more susceptible to ER stress (Figure 5). In this assay, therefore, yeast cells lacking *BXI1* were phenotypically comparable to metazoan cells lacking *BI-1*, which are also sensitive to ER stress inducing drugs.[19]

**Figure 6:**
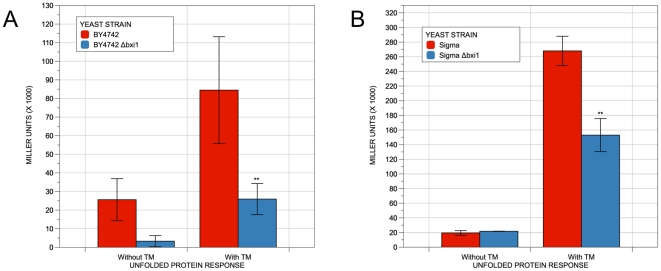
Deleting *BXI1* diminishes the unfolded protein response induced by tunicamycin. Cells of the indicated genotype were transformed with a UPR-lacZ reporter, grown overnight in selective media at 30°C. They were then resuspended in fresh media and allowed to reach exponential phase (an approximate OD_600_ value of 0.4). Next, they were resuspended in fresh media or in fresh media containing 0.6 µg/ml TM and allowed to grow at 30°C for three hours. β-galactosidase assays with the colorimetric substrate *o*-nitrophenyl-β-D-galactopyranoside (ONPG) were then performed using the permeabilized cell method and quantified as previously described. [37], [41], [42] The differences in β-galactosidase levels were deemed statistically significant by the Student's t-test (p<0.05). Error bars indicate standard deviations for trials with at least three independent transformants.

To see if the sensitivity of the *Δbxi1* cells to BME and TM could be explained by changes in the yeast cells' unfolded protein response, we transformed wildtype and mutant yeast cells from both strain backgrounds with pMCZ-Y, a plasmid containing a UPR-responsive UPRE-lacZ reporter [35], and determined the levels of the UPR in both uninduced and induced conditions. As shown in Figure 6, cells lacking *BXI1* had a significantly diminished response to TM induction of the UPR, again, regardless of strain background. In contrast, no significant difference in the uninduced levels of the UPR was observed between wildtype and mutant cells suggesting that the observed differences in the UPR could be attributable to the diminished ability of *Δbxi1* cells to mount a response to ER stress rather than to differences in the underlying basal UPR itself. Thus, like its metazoan counterparts, *BXI1* appears to be involved in the unfolded protein response. Given this finding, it is not clear why a previously published report reported no link between *BXI1* function and the UPR in yeast. [23] One possibility is the difference in strain backgrounds: Lisbona et al. were testing cells in the W303 strain background [23] while our assays were done with cells in the BY4742 and the Σ1278b strain backgrounds. Nonetheless, given the reproducibility of our results in two unrelated strain backgrounds, we are confident that *BXI1* function is linked to the unfolded protein response in yeast.

**Figure 7:**
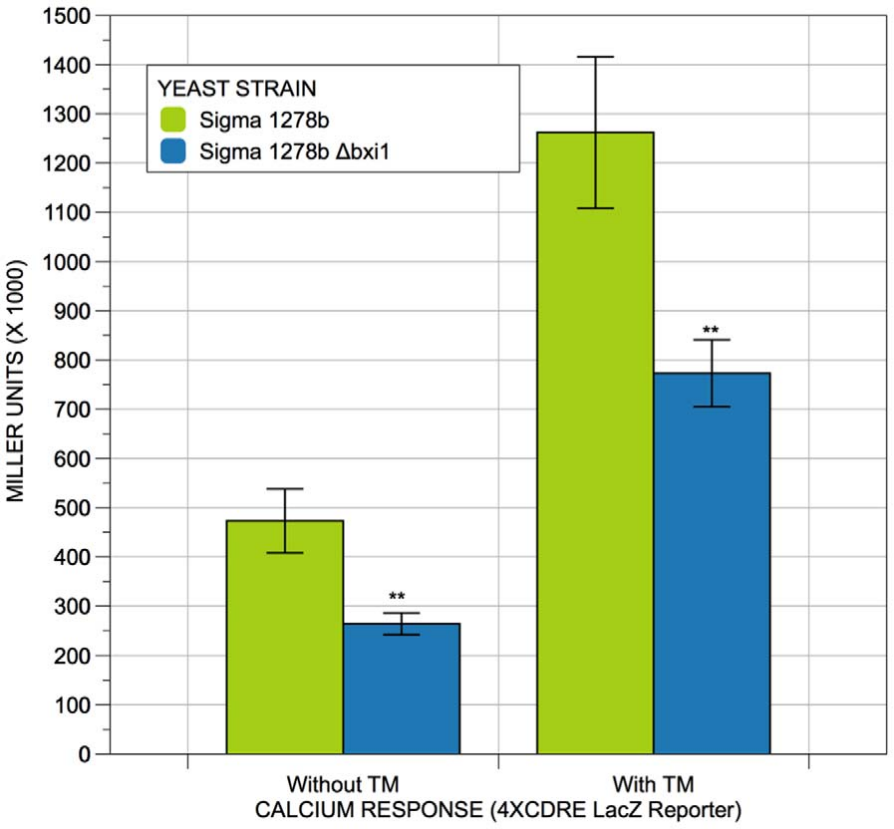
Deleting *BXI1* diminishes calcium signaling in the absence and presence of unfolded proteins in the ER. Cells of the indicated genotype in the Σ1278b strain background were transformed with a 4XCDRE-lacZ reporter, grown overnight in selective media at 30°C. They were then resuspended in fresh media and allowed to reach exponential phase (an approximate OD_600_ value of 0.4). Next, they were resuspended in fresh media or in fresh media containing 0.6 µg/ml TM and allowed to grow at 30°C for three hours. β-galactosidase assays with the colorimetric substrate *o*-nitrophenyl-β-D-galactopyranoside (ONPG) were then performed and quantified using the permeabilized cell method as previously described. [37], [41], [42] The differences in β-galactosidase levels were deemed statistically significant by the Student's t-test (p<0.05). Error bars indicate standard deviations for trials with at least three independent transformants.

Finally, given the association between BI-1 function and the regulation of calcium levels in mammalian cells [24], [47], we decided to uncover any links between *BXI1* function and the calcium signaling pathways in yeast. To accomplish this, we transformed plasmid pAMS366, a high copy plasmid containing a 4XCDRE-lacZ reporter [36] into both wildtype and *Δbxi1*cells and cultured the cells in media with and without tunicamycin. The CDRE (calcineurin-dependent response element) is a promoter element that supports calcineurin-dependent transcriptional induction in response to Ca^2+^. [36] Bonilla et al. have shown that the CDRE-lacZ reporter is activated by the accumulation of misfolded proteins in the ER, suggesting that ER stress is coupled to the calcium influx and signaling pathways in yeast. [48], [49] As shown in Figure 7, we discovered that cells lacking *BXI1* have a diminished calcium signaling response both in the absence and in the presence of unfolded proteins within the yeast endoplasmic reticulum suggesting that these cells may have lower basal levels of calcium. Experiments are underway in our laboratory to uncover the molecular mechanism underlying this link between *BXI1* function in the ER and calcineurin-dependent calcium signaling in the cytoplasm and the nucleus. Nonetheless, our data suggests that like mammalian *BI-1*, yeast *BXI1* may also be involved in regulating calcium levels in the cell more generally and in the ER more specifically.

In conclusion, our study provides evidence that suggests that yeast Bax inhibitor-1, like its metazoan counterparts, is an ER-localized protein that links the unfolded protein response with programmed cell death. There is evidence that a mechanism exists that links ER stress to Ca^2+^ influx and signaling pathways to prevent cell death, and that this mechanism does not require the canonical UPR defined by Ire1p and Hac1p. [49] We are working to determine if Bxi1p could be involved in this novel and still uncharacterized pathway.
